# Treatment of lumbar curves in adolescent females affected by idiopathic scoliosis with a progressive action short brace (PASB): assessment of results according to the SRS committee on bracing and nonoperative management standardization criteria

**DOI:** 10.1186/1748-7161-7-S1-O26

**Published:** 2012-01-27

**Authors:** AG Aulisa, V Guzzanti, C Perisano, F Falciglia, G Maggi, L Aulisa

**Affiliations:** 1 Children Hospital Bambino Gesù, Rome, Italy; 2 A. Gemelli” Hospital, Università Cattolica del Sacro Cuore, Italy; 3 A. Gemelli Hospital, Università Cattolica del Sacro Cuore, Italy

## Background

The effectiveness of conservative treatment of scoliosis is controversial. Some studies suggest that brace is effective in stopping curve progression, whilst others did not report such an effect.

The purpose of the present study was to effectiveness of PASB in the correction of lumbar curves, in agreement with the SRS Committee on Bracing and Nonoperative Management Standardisation Criteria [1][2].

## Materials and methods

Fourty adolescent females (mean age 12.95 ± 1.72 years) with lumbar curve and a pretreatment Risser score ranging from 0 to 2 have been enrolled. The minimum duration of follow-up was 24 months (mean: 41.75 ± 34.47 months). Antero-posterior radiographs were used to estimate the curve magnitude (CM) and the torsion of the apical vertebra (TA) at 5 time points: beginning of treatment (t1), one year after the beginning of treatment (t2), intermediate time between t1 and t4 (t3), end of weaning (t4), 2-year minimum follow-up from t4 (t5). Three situations were distinguished: curve correction, curve stabilisation and curve progression.

## Results

CM mean value was 26.43 ± 2.77 SD at t1 and 13.80 ± 7.94 SD at t5. TA was 10.83 ± 3.74 SD at t1 and 7.88 ± 4.24 at t5. The variation between measures of Cobb and Perdriolle degrees at t1,2,3,4,5 and between CM t5-t1 and TA t5-t1 were significantly different.

Curve correction was accomplished in 82.5% of patients, whereas a curve stabilisation was obtained in 17.5% of patients.

## Conclusions

The PASB, due to its peculiar biomechanical action on vertebral modelling, is highly effective in correcting lumbar curves.
